# Vegetable Cost Metrics Show That Potatoes and Beans Provide Most Nutrients Per Penny

**DOI:** 10.1371/journal.pone.0063277

**Published:** 2013-05-15

**Authors:** Adam Drewnowski, Colin D. Rehm

**Affiliations:** Center for Public Health Nutrition, School of Public Health, University of Washington, Seattle, Washington, United States of America; Old Dominion University, United States of America

## Abstract

Vegetables are important sources of dietary fiber, vitamins and minerals in the diets of children. The United States Department of Agriculture (USDA) National School Lunch Program has new requirements for weekly servings of vegetable subgroups as well as beans and peas. This study estimated the cost impact of meeting the USDA requirements using 2008 national prices for 98 vegetables, fresh, frozen, and canned. Food costs were calculated per 100 grams, per 100 calories, and per edible cup. Rank 6 score, a nutrient density measure was based on six nutrients: dietary fiber; potassium; magnesium; and vitamins A, C, and K. Individual nutrient costs were measured as the monetary cost of 10% daily value of each nutrient per cup equivalent. ANOVAs with post hoc tests showed that beans and starchy vegetables, including white potatoes, were cheaper per 100 calories than were dark-green and deep-yellow vegetables. Fresh, frozen, and canned vegetables had similar nutrient profiles and provided comparable nutritional value. However, less than half (n = 46) of the 98 vegetables listed by the USDA were were consumed >5 times by children and adolescents in the 2003–4 National Health and Nutrition Examination Survey database. For the more frequently consumed vegetables, potatoes and beans were the lowest-cost sources of potassium and fiber. These new metrics of affordable nutrition can help food service and health professionals identify those vegetable subgroups in the school lunch that provide the best nutritional value per penny.

## Introduction

The 2010 Dietary Guidelines for Americans (DGA) [1] have emphasized how important vegetables are to a healthy diet. Eating a wide variety of vegetables is a good way to improve dietary nutrient density, without consuming excess calories [1]. In the United States Department of Agriculture’s (USDA) ChooseMyPlate application, half of the recommended plate is composed of vegetables and fruit [2].

The National School Lunch Program has established weekly requirements for total vegetables as well as for vegetable subgroups: dark green, orange, starchy, and “other” vegetables [3] as well as for beans and peas (legumes). Fresh, frozen, and canned products are allowed to make up the 5-cup weekly total [3]. This recommendation, intended to capitalize on the nutrient content of different vegetables, comes at a price since some vegetables are significantly more expensive than others [4]–[6]. Furthermore, not all vegetables are equally accepted by schoolchildren [7]. School food services would benefit from knowing which vegetables are both acceptable and provide the most nutrients per unit cost.

Vegetables are foods of high nutrient density and relatively low energy content [4]. The present research goal is to compare fresh, frozen, and canned vegetables in terms of nutrients per calorie and nutrients per penny. Our analyses, designed to follow the USDA guidelines on school lunches [3], take into account the frequency of consumption of different vegetables in the United States. Ideally, vegetables served at school lunch ought to be nutrient-dense, affordable, and appealing.

Providing healthier school meals without increasing costs poses a challenge to school food services [8], [9]. Meals built around vegetables and fruit are nutrient-rich but tend to cost more per calorie than do meals that are energy-dense but nutrient poor [10], [11]. There have also been concerns that some vegetables simply do not provide sufficient calories, being 90% water [4]. How to measure vegetables prices has been another topic of debate [12]–[14]. On one hand, food prices per gram do not reflect the high water content of some vegetables and salad greens. On the other hand, food prices per calorie mask the fact that some vegetables are more nutrient-rich. Nutrition economics could benefit from better tools [4]. In the present analyses we developed a new metric of nutrients per unit cost.

Measures of affordable nutrition should help identify those vegetables that provide the best nutritional value per penny and are well-accepted by schoolchildren [8]. Previous studies have shown that some vegetables can be low-cost sources of several key nutrients [4], [10], including potassium, fiber, and vitamin C. In the present study, vegetables that provide the best nutritional value at an affordable cost were identified using a combination of nutrient profiling methods [15] and national food prices data [16], [17].

## Methods

### The Nutrient and Food Price Databases

#### The nutrient composition database

The USDA Food and Nutrient Database for Dietary Studies 2.0 (FNDDS 2.0) is used to code, process, and analyze the What We Eat in America food intake data for 2003–4 [18]. The files include detailed food descriptions for >6,940 foods from all food groups, typical food portions and weights, method of preparation (where available), and nutrient values for energy and 60 nutrients. Each food is identified by a unique 8-digit code, where the first digit identifies the major food group. The second digit identifies subgroups (white potatoes, dark green vegetables, deep yellow vegetables, tomatoes, and other vegetables), whereas the third and subsequent digits provide ever-finer discrimination down to the individual food item. The FNDDS 2.0 database also specifies whether the vegetables were consumed cooked or raw and whether they were cooked from fresh, frozen or canned. Canned and bottled vegetable juices and vegetable soups are included in the vegetables group. The FNDDS 2.0 database generally does not provide brand names and the vast majority of items in the vegetable group are generic [18]. This version of FNDDS was chosen because MyPyramid Equivalents matching the database were available. Such data were not available with newer versions of FNDDS, including those that correspond to more recent National Health and Nutrition Examination Survey (NHANES) cycles.

#### The food price database

Fruit and vegetable prices were based on the 2011 Economic Research Service and USDA report [16], which used 2008 Nielsen Homescan data to calculate the average price of a pound (or, for juices, a pint) of 153 fresh and processed fruits and vegetables at retail stores. In order to estimate price per edible cup equivalent for each food, retail quantities were adjusted for the removal of inedible parts and cooking that occur prior to consumption [18]. For example, 1 pound of store-bought fresh pineapple yields 0.51 pound of edible pineapple. Data from the USDA National Nutrient Database for Standard Reference (Release 21) and USDA’s Food Yields Summarized by Different Stages of Preparation were used to estimate edible weights. The MyPyramid Equivalents Database, 2.0 was used to define edible cup equivalents.

The 2008 USDA fruit and vegetables prices were merged with the FNDDS 2.0 nutrient composition database. Food and Drug Administration serving sizes were based on Reference Amounts Customarily Consumed (RACC).

### The Rank 6 Affordability Index

The 2010 DGA identified vegetables as excellent sources of six nutrients: dietary fiber; potassium; magnesium; and vitamins A, C and K [1]. For each of these nutrients, we estimated the % daily value (% DV) provided per serving for each vegetable in the database [15]. This value can be used to identify the most nutrient-rich sources of a single nutrient. An index-based measure was then developed, based on the median ranking of the nutrient density of these six nutrients, with a minimum possible score of 1 and a maximum possible score of 98. This rank-based measure was then divided by cost to create a new affordability measure, with higher values representing greater ranking of these 6 target nutrients per unit cost. Rankings were used as opposed to absolute values because many vegetables contained no amount of any nutrients, particularly for vitamin A.

Additional analyses graphically identified the foods providing the most potassium and fiber. These nutrients were selected because prior analyses suggested that they were nutrients most sensitive to diet costs [19]. In addition, they were identified by the 2010 Dietary Guidelines as nutrients of public health concern in the American diet [1].

### Frequency of Consumption

For maximum nutritional benefits, the vegetables that are offered at school lunch by the food service ought to be accepted and eaten by schoolchildren. For each of the 98 vegetables with 2008 prices provided by the USDA, we ascertained the frequency of consumption (days 1 and 2) from food listings in the NHANES 2003–04 database among children and adolescents age 5–14, the age groups most likely to participate in the school-lunch program. Because some foods include many similar items in the NHANES data, foods were combined (e.g., the nutrient database used in NHANES 2003–04 includes 12 types of canned corn, so the sum of frequency of consumption for these items were included). The listed frequency of consumption was merely frequency of use and did not reflect portion size. For example, raw tomatoes appeared in the database 3,391 times, most likely as garnish, whereas tomato juice appeared 38 times. However, the frequency measure does indicate whether a given vegetable is a part of mainstream food habits or not. In the present study, NHANES frequency of use was a measure of presumed acceptance and availability.

### Statistical Analyses

Analyses were performed using the Statistical Package for the Social Sciences (SPSS) version 11.0 and Stata 11.2. One-way analyses of variance and comparisons between means (where indicated) were the principal analyses performed. We evaluated heteroskedacity graphically and with the Breusch-Pagan Test. If there was evidence of heteroskedacity we estimated the robust error variance using the method described by Davidson and MacKinnon [20]. An alpha-level of 0.05 was used to determine statistical significance.

## Results

### Vegetable Categories

Mean energy density, water content, and relative prices per 100 g, per 100 kcal, and per serving for 5 vegetable subgroups are shown in **Table 1**. It can be seen that dark green vegetables (including leafy greens) had the highest mean water content (91%) and the lowest energy density. By contrast, beans and peas, followed by starchy vegetables, had a lower water content and higher energy density. The 5 USDA vegetable subgroups differed in terms of energy density, water content, price per 100 g, per 100 kcal, and per edible cup (P<0.001).

**Table 1:** Characteristics and prices of 98 items in the vegetable food group in the FNDDS 2.0^1^ nutrient composition database using ERS 2008^2^ prices.

Vegetable subgroups	# of foods	# of times consumed	Energy density	Water content	Price per 100 g	Price per cup	Price per 100 kcal	Rank 6 Score^3^	Rank 6/Cost^4^
	N	N	kcal/100 g	g/100 g	$/100 g	$/cup	$/100 kcal		
**School lunch subgroups**									
Dark-green vegetables	19	171	25 (6.1)	91 (1.8)	0.46 (0.22)	0.67 (0.36)	1.98 (1.13)	67 (13)	112 (33)
Red & orange vegetables	11	327	54 (44.9)	86 (9.1)	0.35 (0.18)	0.63 (0.42)	0.84 (0.49)	53 (20)	103 (52)
Starchy vegetables	10	1,186	89 (23.4)	76 (7.1)	0.32 (0.16)	0.53 (0.26)	0.37 (0.16)	51 (11)	112 (45)
Other vegetables	46	1,710	31 (26.1)	91 (4.7)	0.58 (0.29)	0.91 (0.52)	2.40 (1.34)	39 (19)	52 (32)
Beans & peas (legumes)	12	218	132 (17.8)	66 (8.6)	0.16 (0.08)	0.27 (0.13)	0.12 (0.06)	54 (6)	240 (101)
P-difference	–	<0.001	<0.001	<0.001	<0.001*	<0.001*	<0.001*	<0.001*	<0.001*
**Preparation subgroups** 5									
Vegetables, fresh, raw	19	1,768	30 (32.6)	92 (5.1)	0.54 (0.25)	0.65 (0.37)	2.47 (1.34)	33 (19)	60 (43)
Vegetables fresh, ckd	20	247	39 (29.3)	88 (7.7)	0.56 (0.27)	0.89 (0.49)	2.02 (1.29)	54 (17)	81 (52)
Vegetables, frozen	23	1,172	51 (40.6)	86 (9.1)	0.5 (0.29)	0.85 (0.52)	1.61 (1.52)	58 (14)	89 (41)
Vegetables, canned	24	207	35 (24.4)	90 (5.2)	0.4 (0.24)	0.71 (0.46)	1.52 (1.08)	46 (22)	82 (47)
P-difference^6^	–	<0.001	0.34*	0.038*	0.17	0.29	0.073	<0.001	0.22
**Totals**									
Total vegetables	86	3,394	39 (32.6)	89 (7.3)	0.50 (0.27)	0.78 (0.47)	1.87 (1.34)	48 (21)	96 (78)
Total vegetables & beans	98	3,612	51 (43.6)	86 (10.1)	0.45 (0.28)	0.71 (0.47)	1.65 (1.38)	48 (20)	79 (46)

* Indicates robust test was used, as some evidence of heteroskedascity was present (p<0.10 Breusch-Pagan test for heteroskedasticity). Data are means and standard deviations by United States Department of Agriculture (USDA) lunch regulation subgroup.

1 USDA Food and Nutrient Database for Dietary Studies 2.0.

2 Economic Research Service.

3 Higher scores indicative of higher nutrient density.

4 Higher values represent greater amounts of 6 target nutrients per cost.

5 Data for beans and peas are not repeated here.

6 P-value of difference for each outcome does not include beans or peas and is based on a sample size of 86 foods.

The USDA vegetable subgroups also differed significantly in terms of their nutrient density and nutrient cost (P<0.001). The Rank 6 score of nutrient density and the Rank 6 score divided by cost showed a significant main effect of USDA vegetable subgroups. Although dark green vegtables had the highest nutrient density scores, after accounting for cost, dark-green vegetables (Rank 6 affordability score 112), starchy vegetables (112) and beans (240) provided better nutritional value for money.

By contrast, fresh, frozen, and canned vegetables were not significantly different from each other in cost, though there was some indication of a difference by nutrient density, with raw uncooked vegetables having significantly lower nutrient density than fresh cooked vegetables. Fresh cooked, frozen and canned vegetables were comparable in terms of nutrient density and no differences were observed by the affordability metric for these vegetable groups.

### Frequency of Consumption

Data in **Table 1** show that the relative frequency of consumption varied widely across vegetable subgroups. The USDA vegetable prices database included 98 whole vegetables, of which 81 were consumed at least once, and 46 were consumed at least 5 times, while 17 were not consumed by any children and adolescents, including frozen mustard greens, canned kale, fresh cooked kale, canned turnip greens, frozen turnip greens, canned mustard greens, frozen winter squash, canned lima beans, canned potatoes, canned okra, frozen artichoke, canned summer squash, fresh artichoke, fresh cooked brussels sprouts, canned whole mushroom slices, frozen okra and canned artichoke.

The most frequently consumed vegetables were French fried potatoes, iceberg lettuce, and raw tomatoes. Once the acceptance criterion was applied, the number of dark green and red and orange vegetables on the USDA list dropped from 30 to 12, whereas the number of “other” vegetables dropped from 46 to 21.

To make the present analyses directly applicable to the school lunch situation, further analyses were restricted to those vegetables that were consumed >5 times in the NHANES database. Those 46 vegetables, divided into USDA school lunch vegetable subgroups, are presented in **Table 2**, together with their frequencies of consumption.

**Table 2:** Median price ($) per 10% Daily Value (DV) by school lunch vegetable category.

Vegetable subgroup	N	Potassium	Dietary fiber	Magnesium	Vitamin C	Vitamin A	Vitamin K	Rank 6 Score	Rank 6/Cost
Dark-green^2^	6	0.54	0.32	0.60	0.12	0.30	0.17	67.5	108.0
Red/orange^3^	5	0.34	0.20	0.97	0.26	0.05	1.89	43.0	107.5
Starchy – all^4,5^	7	0.49	0.24	0.46	0.17	8.23	8.41	47.5	120.9
Starchy - Potatoes^4^	2	0.14	0.19	0.29	0.10	–	6.64	49.5	177.9
Starchy - excludes potatoes^5^	5	0.51	0.28	0.58	0.20	4.85	–	47.5	102.7
Beans/peas^6^	7	0.10	0.05	0.10	0.58	–	3.70	48.5	257.5
Other^7^	21	0.81	0.49	1.12	0.34	2.48	2.93	36.5	48.2

Analysis restricted to vegetables and beans listed 5 or more times in 2003–4 NHANES^1^ database.

1 National Health and Nutrition Examination Survey.

2 Broccoli florets, fresh, cooked (41); Broccoli, frozen (41); Spinach, fresh-cut, fresh, raw (40); Romaine lettuce, fresh (19); Collard greens, frozen (8); Spinach, frozen (7).

3 Carrots, baby, fresh, raw (138); Carrots, whole, fresh, raw (138); Carrots, frozen (20); Sweet potatoes, fresh, cooked (12); Sweet potato fries, frozen (5);

4 Potatoes, frozen, French fries (838); Potatoes, fresh, cooked (79).

5 Corn, sweet, whole kernel, canned (104); Corn, sweet, whole kernel, frozen (104); Peas, green, frozen (29); Corn, sweet, fresh, cooked (23); Peas, green, canned (7).

6 Pinto beans, dried (65.5); Pinto beans, canned (65.5); Black beans, dried (16.5); Black beans, canned (16.5); Lentils, dried (14); Red Kidney beans, canned (11); Red kidney beans, dried (11).

7 Iceberg lettuce, fresh, raw (708); Tomatoes, cherry, fresh, raw (151); Tomatoes, roma, fresh, raw (151); Tomatoes, round, fresh, raw (151); onions, fresh, raw (146); Cabbage, fresh, cooked (54); Green beans, whole, frozen (40); Green beans, cut, frozen (40); Green beans, cut, canned (31); Avocados, fresh, raw (31); Green beans, whole, canned (31); Brussels sprouts, frozen (23); Celery stalks, fresh, raw (22); Celery hearts, fresh, raw (22); Green peppers, bell, fresh (20); Radishes, fresh, raw (13); Red peppers, bell, fresh (12); Olives, black, pitted, canned (11); Asparagus, fresh, cooked (11); Tomatoes, canned (6); Squash, summer, frozen (5).

### Lowest Nutrient Costs Per Penny

**Figure 1:**
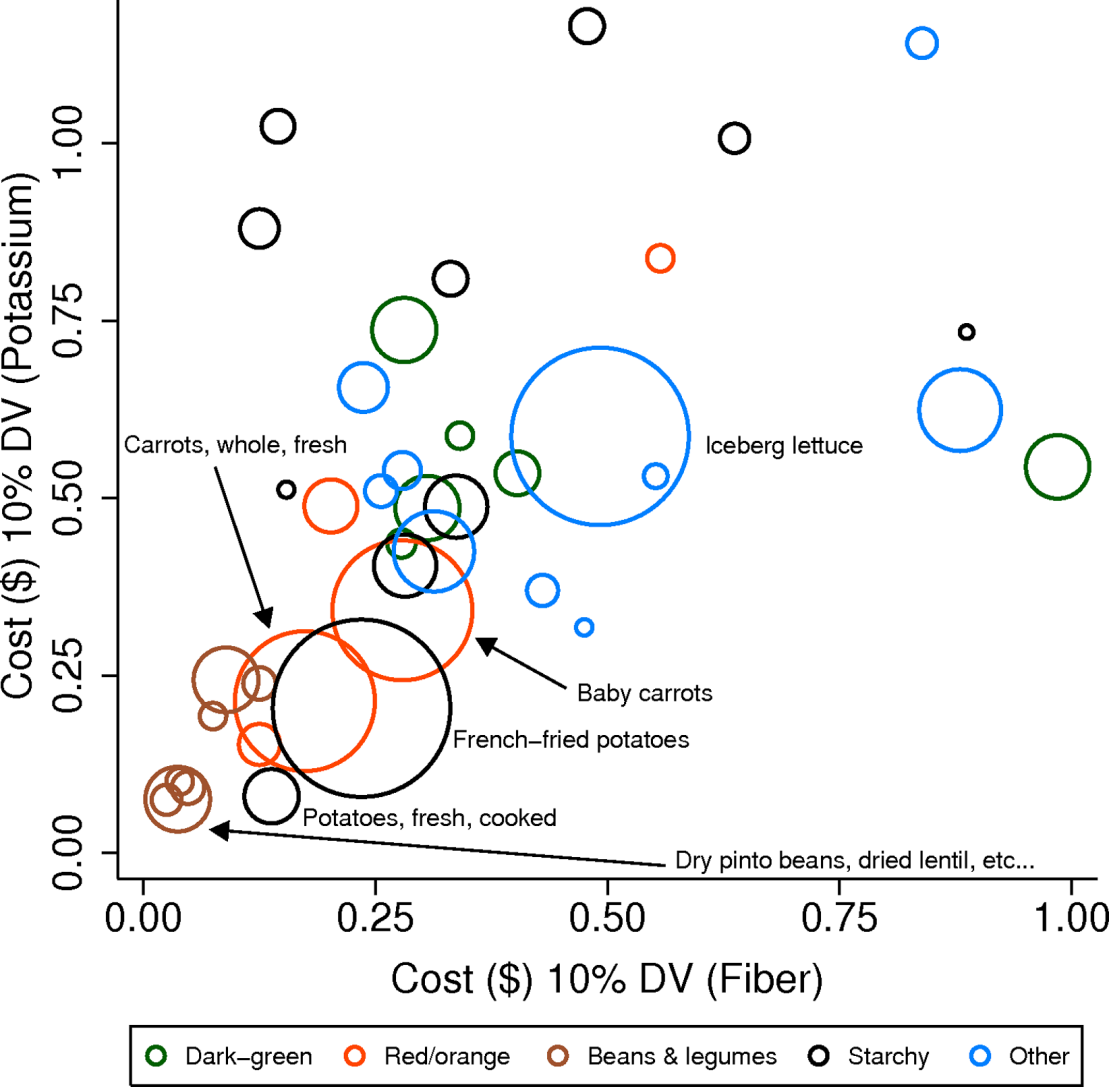
The relative cost for 10% Daily Value for potassium and fiber, including frequency of consumption. Size of circle corresponds to frequency of consumption by children and adolescents, age 5–14.

**Figure 1** provides a graphic representation of the monetary costs of obtaining 10% DV of fiber (x-axis) and potassium (y-axis) from individual vegetables and from beans and peas. Items closest to the origin are those that provide these nutrients at the lowest cost. It can be seen that the lowest-cost items were beans (pinto beans and lentils), white potatoes, sweet potatoes, French fried potatoes, and carrots. Of these, beans were least expensive but also provided the most calories per gram.

**Table 2** identifies the median price per 10% DV for the 6 nutrients of interest by the USDA school lunch vegetable category. Because of the differences identified in **Figure 1**, starchy vegetables are presented overall and are further disaggregated to include potatoes and non-potatoes. The median cost per 10% DV for potassium and fiber was lowest for potatoes ($0.14 for potassium and $0.19 for fiber) and beans ($0.10 and $0.05). For vitamin C, potatoes ($0.10) and dark-green vegetables ($0.12) had the lowest cost per 10% DV. For vitamin A and K, dark-green vegetables had the lowest cost per 10% DV. The combined affordability metric showed that beans had the highest median value (257.5), followed by potatoes (177.9). Individual vegetables, including white potatoes, and different varieties of beans that were the lowest-cost sources of specific nutrients are further identified in **Table 3**.

**Table 3:** Median and mean price per 10% Daily Value (DV) of single nutrients among vegetables consumed 5 or more times.

Nutrient	n	Median price per 10% DV	Mean price per 10% DV	Five lowest-cost options (cost per 10% DV)
Dietary fiber	46	0.31	0.43	Lentils, dried ($0.03); Pinto beans, dried ($0.04); Red kidney beans, dried ($0.04); Black beans, dried ($0.05); Red Kidney beans, canned ($0.08)
Potassium	46	0.53	1.23	Lentils, dried ($0.08); Pinto beans, dried ($0.08); Potatoes, fresh, cooked ($0.08); Black beans, dried ($0.09); Red kidney beans, dried ($0.10)
Magnesium	46	0.77	1.04	Pinto beans, dried ($0.06); Black beans, dried ($0.08); Lentils, dried ($0.09); Red kidney beans, dried ($0.10); Potatoes, fresh, cooked ($0.17)
Vitamin A	36	1.61	3.09	Carrots, whole, fresh, raw ($0.04); Sweet potatoes, fresh, cooked ($0.04); Carrots, frozen ($0.05); Carrots, baby, fresh, raw ($0.06); Collard greens, frozen ($0.08)
Vitamin C	44	0.28	0.43	Red peppers, bell, fresh ($0.03); Broccoli florets, fresh, cooked ($0.04); Green peppers, bell, fresh ($0.04); Broccoli, frozen ($0.05);Cabbage, fresh, cooked ($0.05)
Vitamin K	45	2.93	10.29	Collard greens, frozen ($0.04); Spinach, frozen ($0.08); Spinach, fresh-cut, fresh, raw ($0.14); Brussels sprouts, frozen ($0.17);Romaine lettuce, fresh ($0.19)

**Figure 2:**
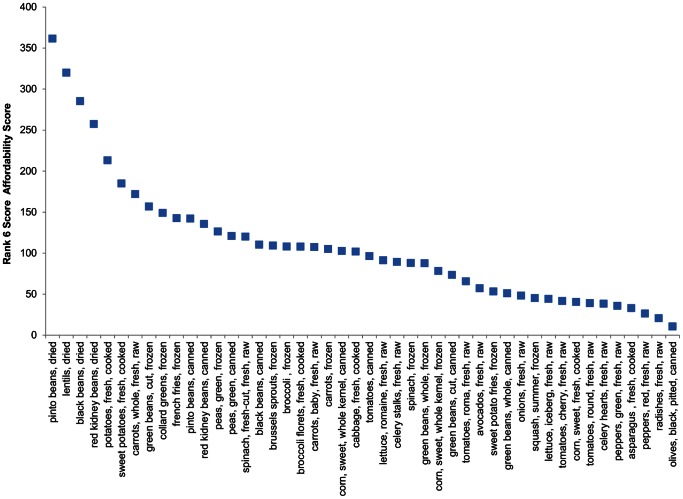
Rank 6– Nutrient Density Score among vegetables consumed 5 or more times.

**Figure 2** ranks the 46 acceptable vegetables according to the composite Rank 6 affordability index. Overall, the best nutritional value was provided by beans, white potatoes, sweet potatoes, and carrots. Of the vegetables with the highest affordability scores, white potatoes (fried and non-fried) and carrots had the highest frequency of use. It is also worth noting that not all of the top-ranked products were fresh; frozen and canned produce also benefited from high affordability scores, specifically canned beans, green beans and collard greens.

## Discussion

To manage nutrition and costs, vegetables served as part of the school lunch ought to be nutrient dense, affordable, easy to prepare and serve, and appealing to children. The present analyses combined nutrient density, cost, and frequency of use to create new affordability metrics. Vegetables provide some key vitamins and minerals at a relatively low cost, as described in prior research [4], [10].

Nutrient density measures were based on 6 nutrients. Based on Dietary Guidelines to Americans, vegetables are important sources of fiber, potassium, magnesium, and vitamins A, C, and K in the American diet. The Rank 6 affordability index incorporated a rank-based measure of nutrient density and the cost per standard serving to provide a summary measure of affordability. The individual monetary cost of 10% DV for each of these 6 nutrients was another measure of nutrient cost.

Finally, frequency of use in the 2003–4 NHANES database was the population based indication of a given vegetable’s acceptability. Out of 98 vegetables, only 46 were consumed more than 5 times, led by potatoes, carrots, tomatoes, onion and iceberg lettuce. Clearly, not all vegetables were the same in terms of general appeal and some were more likely than others to be rejected by schoolchildren.

Previous research, based on 2003 national food prices per calorie, per serving, and per gram [10], [13] showed that vegetables were low-cost sources of potassium, vitamin A, vitamin C, and dietary fiber. Lowest-cost fiber was provided by beans and legumes whereas lowest-cost vitamin C was provided by vegetables and fruit [10], [13]. The present study used the most recent USDA vegetable prices for 2008 [16] and followed the recent school lunch regulations to classify vegetables into subgroups.

Improved school lunches need to maximize nutrition and minimize waste while remaining cost neutral [8]. The present analyses identified white potatoes (including fried), sweet potatoes, beans, carrots and some dark green vegetables as both affordable and nutrient-dense. However, not all affordable nutrient dense vegetables are part of mainstream eating habits. The consumption frequency of sweet potatoes and some dark green leafy, and non leafy vegetables was low. Only beans, white potatoes and carrots managed to combine nutrient density, affordability and consumer acceptance. White potatoes rivaled beans in nutrient density and had lower energy density and much higher frequency of use.

Additional and more detailed studies are clearly needed to determine which affordable nutrient-rich vegetables, fresh, frozen, canned or processed are best accepted by school children. For example, there may be standouts among the dark green vegetables and yellow/orange vegetables that combine high nutrient density and excellent value for money. Also, as indicated in **Figure 2**, high nutrient affordability scores were not limited to fresh or raw produce but included vegetables that were frozen and canned.

While countless studies have evaluated nutrient density scores to date [21]–[24], few studies have attempted to combine measures of affordability and nutrient density into a single summary score [25]. To date, the focus has been on evaluations of the affordability of single nutrients [26], [27] or the ratio of a nutrient density and affordability measure (unpublished data). The Affordable Nutrition Index (ANI) is one proposed measure, which is the ratio of the Nutrient Rich Fodos index per standard portion and the cost per standard portion, with higher values reflecting greater nutrient density per cost. The nutrient density affordability measure used here follows a similar approach and is highly correlated with the ANI (r = 0.94). The measure used here is unique in that it is was specifically created to evaluate the nutrient density and affordability of a single food group, whereas the ANI may have greater utility for between food-group comparisons.

This study has some limitations worth noting. First, it is important to note that the prices used here represent the prices experiences by a relatively small number of Americans, and may not reflect those prices actually experienced by consumers or schools, or those in specific regions that may have higher/lower costs [16]. Schools may be able to purchase items for less cost due to volume. However, assuming that bulk purchasing discounts do not vary considerably by vegetable type, the results of this study should hold.

The nutrient density and affordability index used here has some additional limitations. First, the use of a rank-based score assumes that the difference between each subsequent ranking is similar. However, the use of a rank-based score does avoid the problem of extreme values having undue influence on the summary score. Other nutrient density measures are similarly unit-free [21]–[24]. Second, the examination of affordability and nutrient density is dependent on the nutrients used to calculate the nutrient density score. Therefore, results may vary if different nutrients are used. The nutrients used here were specifically identified in the 2010 Dietary Guidelines as important nutrients for vegetables. Lastly, the nutrient density score used here focuses solely on nutrients to encourage, and does not account for fat, sugar or sodium. Both cooked and raw vegetables are often served with added fat and salt. In addition, care should be taken in examining the nutrient affordability of fried products which are more likely to contain added fats and sodium. However, these products are most frequently consumed by children, making their inclusion in this analysis justified. In comparison to previous work, this study only evaluated the affordability within a single food group. Therefore the results of this study can only be used to evaluate the vegetable group, and cannot evaluate the potential place of other food groups, such as fruit, grains, or dairy products, which may be important in the evaluation of potassium, fiber and magnesium.

### Conclusions

School lunches need to balance taste, cost, convenience and nutritional value [28], [29]. Hungry children may opt for foods of high energy density but potentially lower nutritional value. The present calculations, thus far limited to vegetables, illustrate how the econometric approach to nutrient profiling can help identify affordable nutrient-rich foods within each food group. Effective menu planning requires knowledge of federal regulations, nutrient density standards in relation to costs and children’s food preferences. Joining nutrient density profiling with the economics of food choice behavior is a relatively novel area of research.
